# The status and politics of bicycling in the cities of low- and middle-income countries

**DOI:** 10.1038/s44284-025-00367-y

**Published:** 2026-01-20

**Authors:** Smruthi Bala Kannan, Rahul Goel, Ernest Agyemang, Zahidul Quayyum, Srishti Agrawal, Anisur Rahman Bayazid, Jonathan Anjaria, Kavi Bhalla

**Affiliations:** 1 Department of Public Health Sciences, University of Chicago, Chicago, IL, USA; 2 Transportation Research and Injury Prevention Centre, Indian Institute of Technology, Delhi, India; 3 Department of Geography and Resource Development, University of Ghana, Accra, Ghana; 4 BRAC James P Grant School of Public Health, BRAC University, Dhaka, Bangladesh; 5 Department of Anthropology, Brandeis University, Waltham, MA, USA

## Abstract

Bicycling is promoted in low- and middle-income countries to reduce emissions and improve public health. However, transportation policies often replicate built infrastructure logics from high-income countries, with limited attention to local bicyclists’ experiences and contextual realities. Here, drawing on intercept surveys, semi-structured interviews and ethnographic observations in cities of four low- and middle-income countries, we examine the current state of bicycling and user-perceived barriers. We find that bicycling is largely undertaken by low-income adult men, while women and children cycle mainly within neighborhood enclaves. We identify impediments beyond physical infrastructure, including financial systems, maintenance networks, manufacturing and design capacities, and social supports that shape everyday cycling. Using a mobility justice lens, we examine how these bicycling cultures have persisted within automobile-centric urban geographies. We highlight the importance of addressing these evidence, policy and implementation gaps through an ‘ethics of care’ approach, supported by regional health and environmental agencies and grounded in context-sensitive research.

Bicycling is widely promoted as a strategy for reducing the climate impacts of transport and for improving population health owing to reduced emissions and physical activity benefits^1^. The Intergovernmental Panel on Climate Change (IPCC) promotes bicycling as a key strategy to mitigate transport emissions^2^ and the World Health Organization (WHO) lists bicycling infrastructure as a ‘best buy’ for tackling noncommunicable diseases^3^. Air pollution and physical inactivity are leading risk factors for noncommunicable diseases in low- and middle-income countries (LMICs)^4–6^. Nevertheless, automobiles continue to increase and remain aspirational in the cities of LMICs, with limited understanding of pathways to increase bicycling. Through mixed-methods research in four LMIC cities, we show that bicycling cultures persist in LMIC cities primarily among marginalized communities despite limited infrastructure or policy attention, and we examine the relational experiences of bicyclists that reveal the inequities underlying the expanding automobile-centric transport systems. Viewed through a kinopolitical lens, these bicycling cultures reflect deeper social, economic and political dynamics. The locally embedded experiences and relationships of bicyclists offer valuable insights and resources for building more equitable and sustainable mobility systems that prioritize accessibility, safety and social inclusion for all.

Mimi Sheller’s work on mobility justice emphasizes that equitable and sustainable transitions in mobility systems should attend to the relational and multiscalar kinopolitics within which the contemporary urban and climate crisis are embedded^7^. Some cities in high-income countries (HICs) have made large investments in bicycling infrastructures that have shown successful increased bicycling^8,9^, decreased car use, reduced vehicular emissions and improved air quality^10–13^. Following such examples, many LMICs are increasingly investing in bicycling infrastructures^14,15^. While HICs, including those with low cycling rates, negotiate transitions from peak car-centric automobile cultures, LMICs face highly mixed-traffic roads and expanding highway development, alongside pre-existing transportation equity policies and non-elite bicycling cultures.

Literature documents an exclusionary history of bicycling in the cities of HICs, where built environment, policy and cycling infrastructures have contributed to new forms of social exclusion on the roads^7,16–18^. Intersecting factors such as race and socioeconomic status^19^ or age and migration status^20^ further amplify these barriers to bicycling. However, it is notable that in LMICs, bicycles have also been used to equalize transportation through financial and manufacturing infrastructures, particularly to make bicycles affordable. For example, bicycle distribution schemes have aimed to improve the access of girls to bicycles as part of female empowerment efforts, with growing evidence of their success^21,22^ in a larger, highly gender unequal transportation landscape in India^23^.

Jonathan Anjaria argues that recent urban bicycling-oriented policies in LMICs are primarily using built infrastructure-focused logics from HICs, with limited attention to the experiences of their existing bicyclists and the local context^24^. Taking this critique seriously, we conducted intercept surveys, semi-structured interviews and ethnographic observations in New Delhi and Chennai (India), Dhaka (Bangladesh) and Accra (Ghana). Through mixed-methods research, we ask: “Who bicycles in the selected cities? How do they experience and perceive bicycling? What barriers to bicycling do they face?” We synthesize these findings to describe the infrastructures^25^, including physical, financial and human, that shape bicycling in LMIC cities. Through bicyclists’ relational experiences and anxieties, we trace infrastructures that engage in ‘legitimations of speed’ and those that challenge the ‘unjust power relations of uneven mobility’^7^, and identify pathways to foster increases in bicycling.

## Context

**Extended Data Fig. 1:**
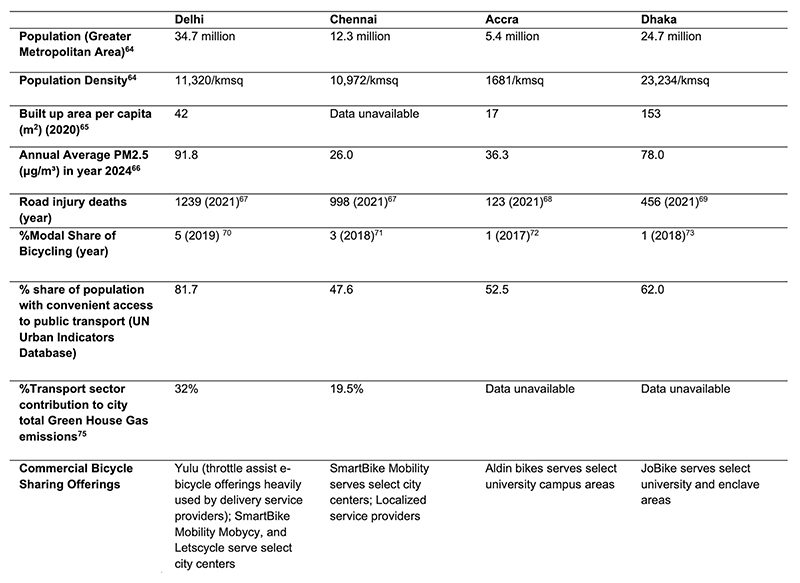
Urban Profiles. The table provides select urban demographic^64,65^, pollution^66^, road injury^67–69^, and transport-related data points^70–75^. The urban data presented here represent approximations derived from heterogeneous sources collected across disparate time frames and purposes, introducing methodological inconsistencies and limitations. The figures in the table should be approached with a consideration of their provisional nature and not be considered from a comparative perspective.

Delhi and Chennai (India), Dhaka (Bangladesh) and Ghana (Accra) are rapidly densifying and expanding cities, with flat terrains and heterogeneous traffic. They share subtropical to tropical climates with very hot summer days and seasonal flooding. Globally, physical inactivity accounts for 7% of all deaths, with the bulk (69% of deaths) occurring in LMICs such as India, Bangladesh and Ghana^4^. Similarly, 12% of global mortality and 19% of cardiovascular mortality are attributable to air pollution^5,6^. The cities share a high burden on people’s health from transport-related pollution^26^, noncommunicable diseases^5,27^ and road traffic injuries (Extended Data Fig. 1). A large shift toward nonmotorized transport, especially bicycling, can help countries toward longer-term climate goals. For example, rapid growth in the motor vehicle fleet (600% in the past two decades) has made transport the fastest-growing contributor to greenhouse gas emissions in India, and transport now accounts for nearly 30% of emissions in the major cities (Extended Data Fig. 1). Among the transport sector options for meeting India’s Nationally Defined Contributions, the largest reductions will be from shifts to low-carbon transport such as walking and bicycling^28^.

Urban planning and strategy documents and discourses in these cities mention bicycling. However, infrastructures to increase bicycling (bicycle-sharing systems and separated lanes) and improve equitable mobility (bicycle distribution through government schemes and phil-anthropic efforts) are conducted with little data on the mode and its user experience. While household bicycle ownership data are available in India in general, limited data are available for the four cities on bicyclist trips, demographics and road use. In this context, we conducted intercept surveys with bicycle users on arterial roads, semi-structured interviews with bicyclists and stakeholders, and ethnographic field observations in diverse locations. Our research explores pathways to support increases in bicycling in such contexts.

## Results

### Who bicycles in Delhi, Chennai, Accra and Dhaka?

#### Socioeconomic status

**Extended Data Fig. 2:**
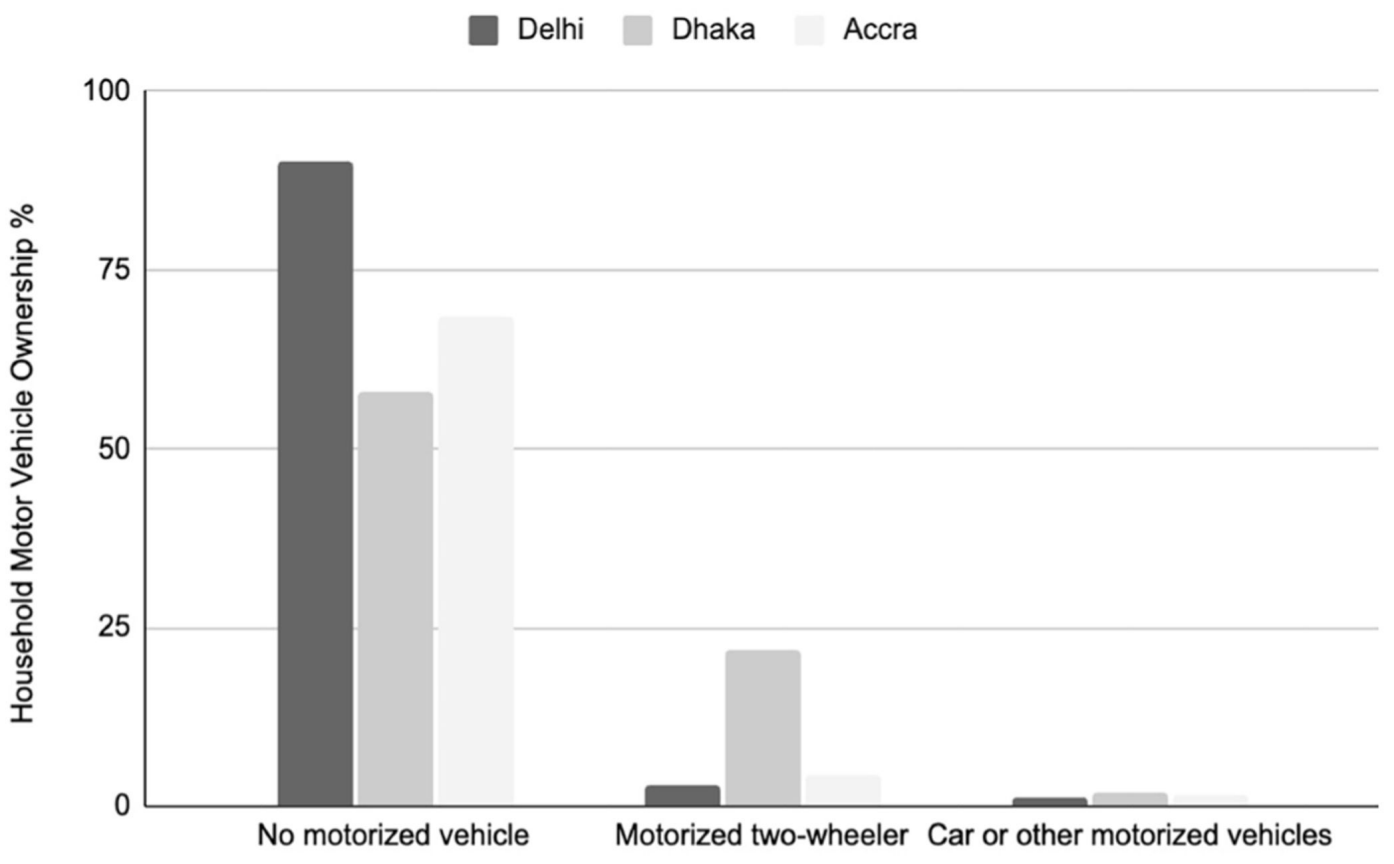
Household Motorized Vehicle Ownership Level. Highlights from Intercept Surveys.

**Extended Data Fig. 3:**
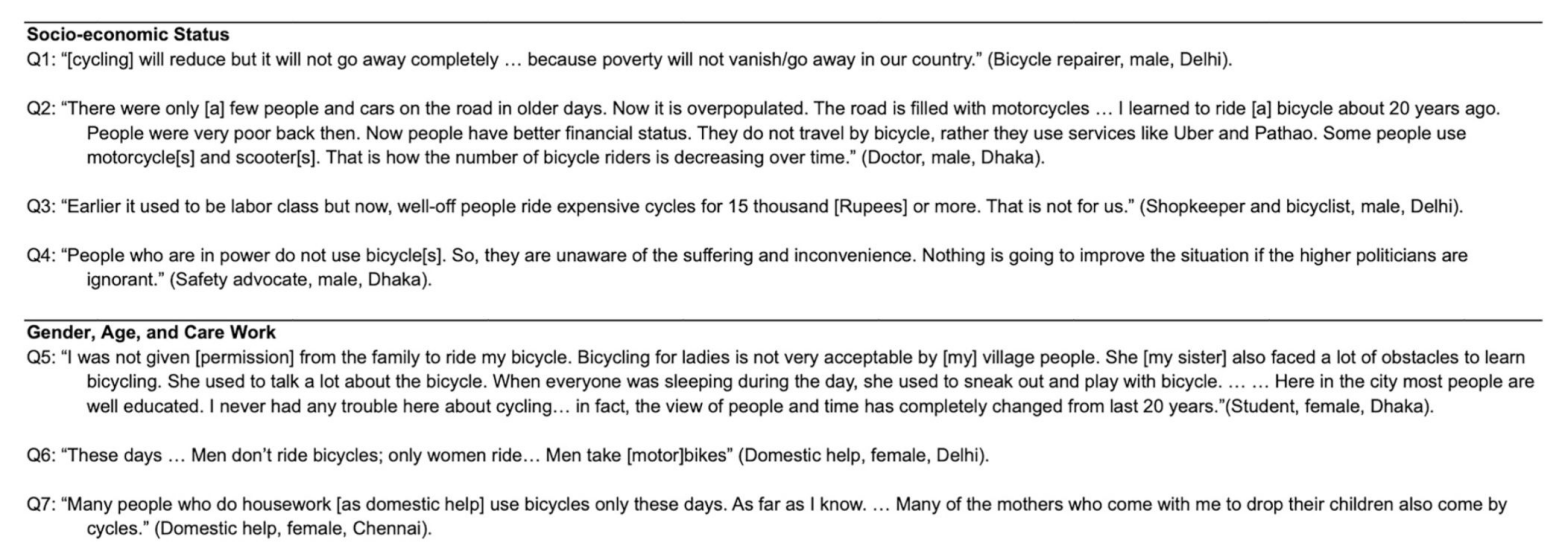
Interview Excerpts: Who bicycles in Delhi, Chennai, Accra, and Dhaka? Excerpts from semi-structured interviews referenced in the sub-section titled ‘Who bicycles in Delhi, Chennai, Accra, and Dhaka?’.

Bicyclists surveyed on arterial roads in Delhi, Dhaka and Accra were largely poor, adult and male. Except for one woman in Dhaka, all survey respondents were men, predominantly young adults to middle aged, with a slightly older distribution in Delhi (Table 1). They were largely from lower-income households in the four cities and had jobs involving substantial physical activity (Table 2). Most respondents’ households owned no other vehicle (Extended Data Fig. 2). Interviewees had a strong tendency to associate bicycling with either poverty or childhood. They noted that once people can afford automobiles, bicycles are not a preferred mode of transportation unless one undertakes bicycling for physical exercise (Extended Data Fig. 3, Q1–2).

Bicycle retailers and activists have emphasized the need for more automobile users to bicycle and for infrastructure to support this transition. Some bicyclists noted an increasing presence of elite bicyclists (Extended Data Fig. 3, Q3), although our surveys and observations showed that they currently comprise a very small proportion of the bicycling population. We observed high volumes of bicycling geographically concentrated in several corridors of the cities at selected times. Notably, policy actors who did not directly work on bicycling typically held the view that there was hardly any bicycling in the cities these days. This invisibility of bicyclists among decision-makers was repeatedly noted by bicyclists and advocates (Extended Data Fig. 3, Q4). The ‘common sense’ view that nobody bicycles was also evident in the limited discussion of bicycling in planning documents, where bicycling was typically mentioned as recreational infrastructure in urban beautification and development projects, and increasingly as active mobility. Sustainability policy documents largely focused on electric vehicles and public transit, with little attention or intentional budgetary allocation to bicycling infrastructure. The school bicycle distribution scheme was a notable exception, enabling adolescents from low-income families to access education. While this policy was implemented in Chennai and several other regions in India^21,22^, all four research cities had philanthropies distributing bicycles for socioeconomic access.

#### Age, gender and care work

Bicycle use varied strongly by age and gender. Children largely cycled within traffic-restricted residential pockets and buildings. The few children we observed on arterial roads were probably of adolescent age, male and were more common in Accra compared to Delhi, Chennai and Dhaka. Interviewees whose adolescent children commuted by bicycle explained that they were either caregivers or needed to travel independently owing to the lack of adult caregivers. Women on bicycles, often as pillion riders, were slightly more common on arterial roads than children. We saw more women cyclists on arterial roads in Chennai than in other cities. A few women cyclists were observed in Delhi and Dhaka on arterial roads, and none in Accra. Some male bicyclists in Delhi expressed reservations about women in their families bicycling. Alternatively, several interviewees in Delhi, Chennai and Dhaka mentioned that their daughters or grand-daughters bicycled but not on larger roads.

We interviewed women bicyclists in residential neighborhoods where domestic workers’ bicycle use was particularly common. They described a hierarchy of vehicles between cycles and automobiles (Extended Data Fig. 3, Q6). Just as automobiles were preferred by higher socioeconomic classes, in households that had both bicycles and motorcycles, men used motorcycles and mentioned traveling longer distances compared to women who used bicycles.

Women interviewees mentioned avoiding arterials and bicycling shorter distances (~2 km) with multiple stops for work and other reasons. By contrast, men typically reported a long single ride to work, even if they trip-chained close to their destination (Table 2). In Chennai and Delhi, women’s travel to work schedules were later in the morning than those of men, who typically reported early morning travel. We observed several women caregivers and students using cycles to carry younger students or multiple school bags (Extended Data Fig. 3, Q7). Many female bicyclists with pillion children and bags dismounted and walked their bicycles when traversing arterial road stretches and resumed bicycling on smaller roads.

Migration status intersected with the gendered experience of urban bicycling. Women bicyclists in Delhi often described migrating from states with active school bicycle distribution schemes, such as West Bengal. A female bicyclist in Dhaka illustrated the complexity, noting that while women’s cycling continued to be a taboo in certain regions, people found workarounds. After moving to the city from her village, she had learned to bicycle as an adult from her younger sister, who had a particular passion for it (Extended Data Fig. 3, Q5). Migration also shaped men’s bicycling. Many survey respondents described learning to bicycle in locations with fewer and slower automobiles on the road compared to their current routes, which they considered risky for their children and younger kin.

### Safety and comfort

#### Sharing roads with automobiles and experiencing risk

**Extended Data Fig. 4:**
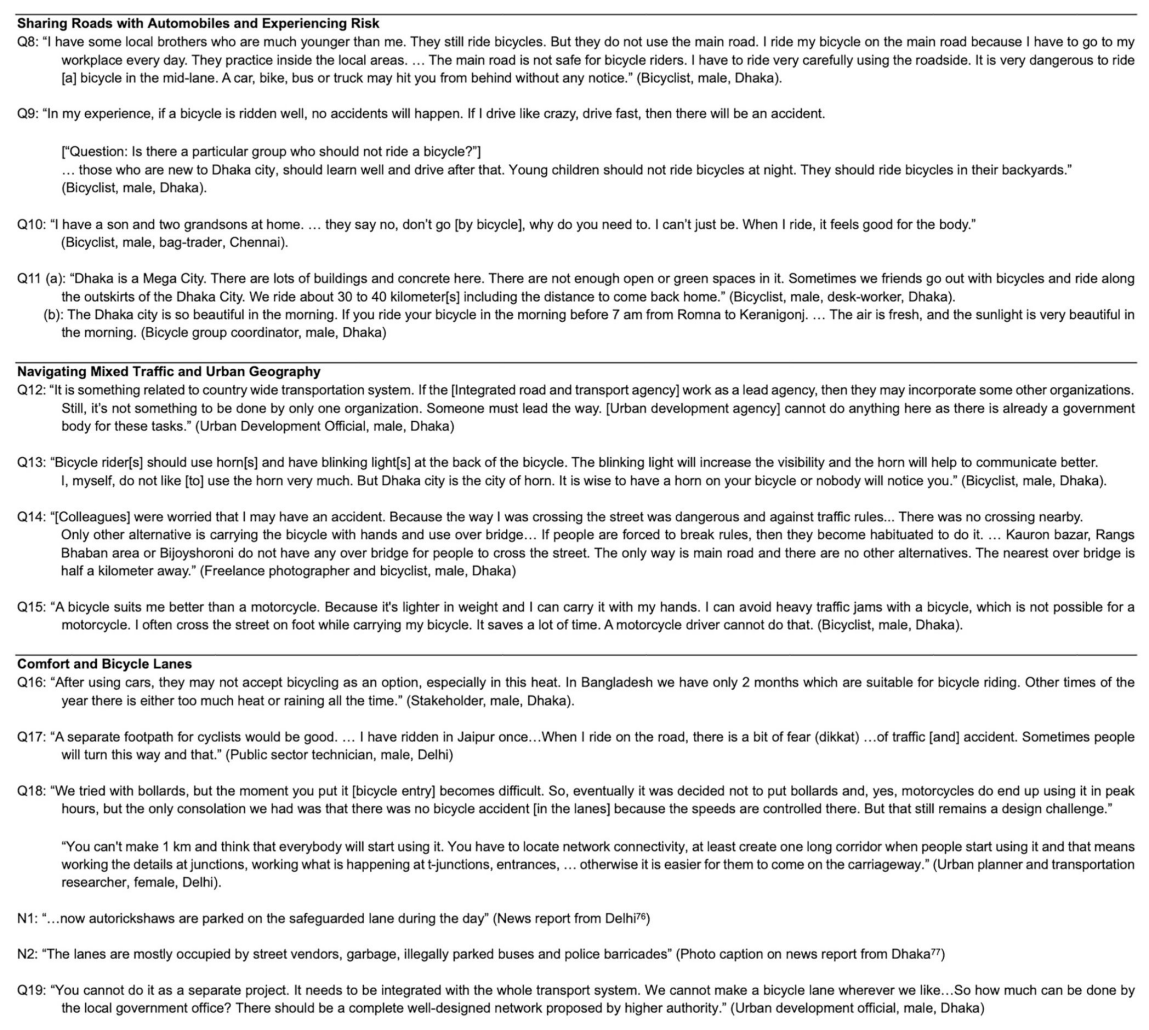
Interview and Media Excerpts: Safety and Comfort. Interview quotations and media excerpts^76,77^: referenced in the sub-section titled ‘Safety and Comfort’.

Bicyclists expressed that arterial roads were unsafe owing to fast-moving vehicular traffic, influencing who can and prefers to ride on such roads. While adult male bicyclists saw themselves as capable of traveling cautiously in high-speed traffic, irrespective of socioeconomic class, they tended to focus on safety risks when describing their younger kin or children riding bicycles (Extended Data Fig. 4, Q8). Many bicyclists and parents placed the responsibility to navigate risks on mixed-traffic roads on individuals, describing children, adolescents and the elderly as incapable of sufficient caution. They described near misses as a routine part of the cycling experience and recollecting minor crashes as a step in learning to bicycle. Crash-related expenditures to repair bicycles or damage to the goods they carried were common.

In describing the dangers associated with bicycling, some interviewees perceived bicycles as particularly unsuitable for persons who might face difficulty recovering from injuries. An interviewee expressed her concern about her diabetic teenage grandson’s safety owing to ‘crazy cycling’ by his peers, worried about his slower healing time if injured. Echoing parents’ anxieties, elderly interviewees—who also mentioned healing slowly—discussed safety and comfort as main reasons why their adult children dissuaded them from bicycling, even when they perceived that bicycling enhanced their well-being (Extended Data Fig. 4, Q10).

While elite and middle-class bicyclists and bicycling activists attributed crashes to automobile drivers or infrastructure, most others placed the onus of safety on the cyclists themselves (Extended Data Fig. 4, Q9). Many elite and middle-class bicyclists who participated in group rides also tended to describe bicycling as a social activity that offered relaxation, ideally outside urban traffic or during early mornings (Extended Data Fig. 4, Q11), while they continued to use motorcycles or cars within the city and to navigate peak automobile traffic.

#### Navigating mixed traffic and urban built environment

We often observed bicyclists interrupted by infrastructure primarily intended to support unimpeded vehicular flow (for example, foot overbridges, flyovers and medians). “[One] can’t cycle there. Now they have fully locked it. Earlier, [you] could easily ride through”, explained a mother who had walked her bike with her young son on the carrier across a busy arterial in Chennai and had climbed a newly built foot-high median to get to the other side.

Medians, U-turns, crossings, intersections and roundabouts, where bicyclists cannot avoid interactions with motorized vehicles by just ‘riding on the side’ or ‘staying in their lane’, were considered particularly risky by parents and adult bicyclists. Bicyclists either waited at pedestrian crossings or grouped to establish their presence before crossing the roads or navigating turns. Infrastructural interruptions increased both the effort and distance for bicyclists. Bicyclists, including adolescent school children, pedaled or walked their cycles up flyovers alongside buses, trucks, cars, motorized three-wheelers and motorcycles. Where pedestrian infrastructures such as overpasses existed, we saw bicyclists carry their bicycles up the stairs. It was also common for bicyclists to subvert such autocentric road rationales by moving counterflow to automobile traffic on the main carriageway, traversing median barriers with their bicycle or holding on to slow-moving automobiles such as autorickshaws to be towed up flyovers (Extended Data Fig. 4, Q14).

While such infrastructural features were a source of anxiety for bicyclists and their kin, they particularly added to the exertion of adult male bicyclists who cycled relatively longer stretches on the ‘main roads’ often designed and maintained by highway authorities for automobiles’ uninterrupted long-distance flow. An urban development official in Dhaka described the lack of clarity on which authority is responsible for supporting increasing bicycling (Extended Data Fig. 4, Q12). We observed this ambiguity across state and metropolitan planning guidelines and budgets.

Bicyclists undertook strategic modifications to make their bicycles more visible in such mixed-traffic roads. Most bicycles we encountered did not have lights and reflectors, and their manufacturer-provided bells got drowned out in the constant automobile honking common in these cities. Bicyclists in Delhi repurposed and used removable road reflectors, which were brighter and more robust than those sold for bicycles. Some bicyclists in Dhaka and Chennai affixed headlights and motorcycle side mirrors that doubled as reflectors. In Dhaka (but not in the other cities), battery-powered horns were used to circumvent this issue (Extended Data Fig. 4, Q13). Such modifications may be read as subversions of automobility through its tools and how bicyclists establish their belonging toward the roads and demonstrate their fluid position between pedestrians and automobiles.

Urban housing geography also shaped bicycling. Adult male survey respondents traveled by cycles to, from and during work for long distances and durations, with the longest commute times in Delhi (Table 1). Respondents often lived far from their work locations, probably because of cheaper housing in the urban periphery, where some had been relocated to resettlement colonies as part of government programs to remove slums near affluent areas^29^.

People bicycled despite these hostile conditions as bicycles were relatively easier to afford and access compared to motor vehicles, especially on routes with limited public transport. The status of bicycles as nonmotorized vehicles, exempt from registration and licensing requirements, reduces barriers to their use and offers unique opportunities for riders on mixed-traffic roads. In Delhi, Dhaka and Chennai, they were also not penalized for riding on footpaths, counterflow to traffic or turning at junctions in ways that are prohibited for motor vehicles. Bicycles also offered benefits in a congested city and were supported in distinctly local ways. Traffic police often took cognizance of bicyclists in Dhaka at signals and allowed them to move ahead of motorized traffic (Extended Data Fig. 4, Q15).

#### Comfort and bicycle lanes

Being exposed to weather conditions and broken road surface conditions were matters of everyday concern. The respondents and interviewees in all four cities described traveling, when possible, early during the day and late in the evening to avoid the harsh sun. Stakeholders saw escaping harsh weather through automobiles as a promise that urban class mobility offered (Extended Data Fig. 4, Q16). However, we witnessed bicyclists, albeit fewer, out during the rain in Delhi, Dhaka and Chennai, who used raincoats, hand-held umbrellas and single-use plastic bags as rain cover. We also encountered bicyclists using the road during extreme heat, including two heatwaves each in Delhi and Dhaka, and flooding in Dhaka during our fieldwork.

Interviewees described segregated pathways with shade as a desirable convenience for bicycling if they were maintained and regulated properly. A public sector technician in Delhi compared a bicycle track in a different city to a segregated pedestrian pathway or ‘footpath’ (Extended Data Fig. 4, Q17). The high number of motorcycles remains a challenge in Delhi, Dhaka and Chennai to bicycle tracks because of their tendency to use the tracks to avoid traffic congestion on the main carriageway. An urban planner and transportation researcher in Delhi shared her experience designing and iterating on a bicycle lane: while speed control measures work well, bicycle lanes pose design challenges because bicycles and two-wheelers are similar in form. Barriers such as bollards that block two-wheelers also impede bicycles (Extended Data Fig. 4, Q18).

We observed, and many interviewees highlighted, that during periods of congestion on the main carriageway, separated bicycle tracks were flooded by motorized two-wheelers, posing a safety hazard and a travel impediment to bicyclists. Numerous news stories echo interviewee responses and document encroachment by motorcycles and street vendors and the disappearance of protective barriers, often within months of installation (Extended Data Fig. 4, N1–N2). Furthermore, it was common for bicycle lanes and tracks to be obstructed by parked cars, shop extensions, fallen trees during the monsoons, and construction debris and rubble. Consequently, we saw bicyclists prefer cruising on the main carriageway of arterials during long commutes, even in the presence of separated bicycle tracks or lanes.

In addition to these issues, experts and activists also emphasized the need for longer lanes or networks throughout the city, rather than providing them in small stretches (Extended Data Fig. 4, Q18–19). The separated bicycle tracks and painted lanes that we observed in Delhi and Dhaka were largely situated near high-income neighborhoods. These tended to be in disconnected and short stretches that would account for an insignificant portion of most bicyclist trips in the area.

### Retail, repair and durability of bicycles

Over two-thirds of respondents in Delhi rode steel-frame fixed-gear roadsters with rod-actuated brakes, 1.25–1.5-inch-thick wheels of varying diameters and fitted with a back carrier. These were also common in the other cities, but fewer than in Delhi (‘Simple’ or ‘Desi cycles’ (Delhi), ‘Saada cycles’ (Chennai), ‘Busanga Volvo’ (Accra) and ‘Phoenix’ or ‘Bangla cycles’ (Dhaka)). Although typically used by poorer bicyclists, these were not the least expensive bicycles but were well-recognized for their durability, longevity and ease of access to parts and repair skills in the market. These roadsters are designed for slow movement and carrying goods or a pillion rider, often for longer distances. Government-distributed bicycles in Chennai targeted at school-going adolescents were these roadsters, which also circulated among the urban public through the second-hand market.

**Extended Data Fig. 5:**
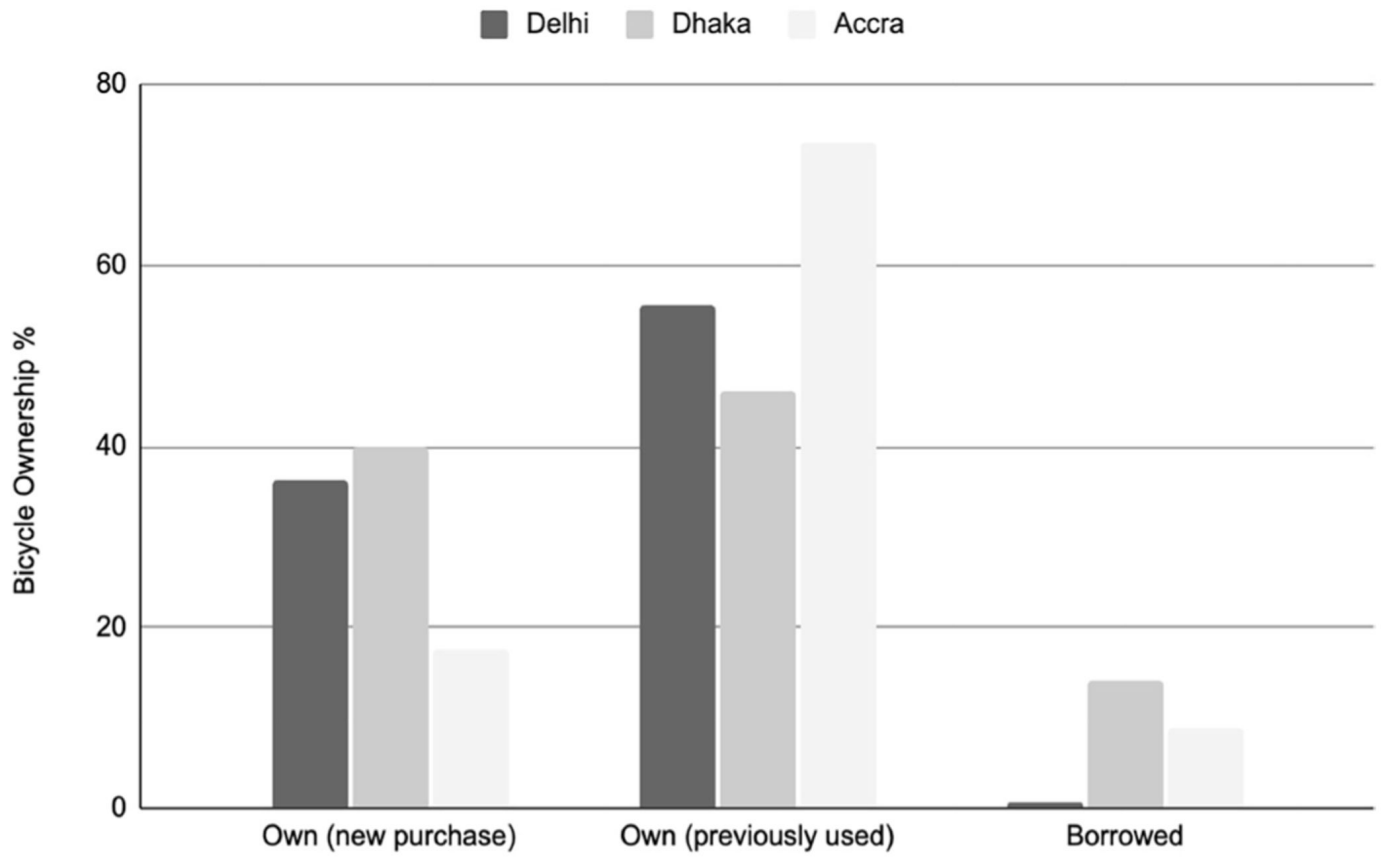
Bicycle Ownership Status. Highlights from the intercept survey data providing the percentage of respondents who owned, through purchase of a new or previously owned bicycle, or had borrowed the bicycle they rode.

**Extended Data Fig. 6:**
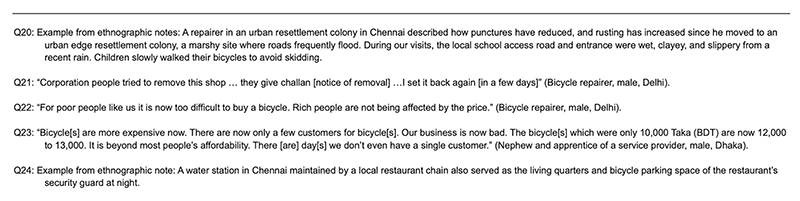
Excerpts from Interview and Ethnographic Notes: Retail, Repair, and Durability of Bicycles. Interview excerpts and ethnographic notes referenced in the sub-section titled ‘Retail, Repair, and Durability of Bicycles’.

Bicycles tended to be used for a long time after purchase (Table 1), including models now phased out of manufacturing. Most respondents rode second-hand bicycles (Extended Data Fig. 5). Bicycle users noted that regular repair and maintenance contributed to their longevity. Across types of stakeholders, people registered the importance of repair shops and services for sustaining bicycling in cities. Repair shop owners tended to be highly knowledgeable about local bicycling conditions and tuned bicycles accordingly (Extended Data Fig. 6, Q20).

Despite their importance to bicycling in the city, repair service providers for bicycles were largely small shops or footpath stalls with a precarious existence. Many had downsized in the past decade and had to frequently negotiate with police and regulatory authorities to continue to occupy footpaths (Extended Data Fig. 6, Q21). The marginal place of the repair service providers further resonated with the marginalization of the low-income bicyclists in the city, as we heard repairers speak of themselves as poor bicycle riders. Larger bicycle retailers shared issues such as increasing taxes on the components and accessories of bicycles that impacted the repair service providers and buyers (Extended Data Fig. 6, Q22–23).

Electric bicycles and bicycle-sharing systems did not connect with such existing bicycle repair providers. We observed very few electric bicycles in the four cities and most repairers, who did not specialize in e-bicycles, expressed difficulties in handling their electrical components and finding parts. Further, existing bicycle-sharing systems in Delhi, Chennai and Dhaka were largely in disuse owing to a lack of maintenance.

Compared to automobiles, bicycle parking required little real estate, and many bicyclists reported parking inside their homes. Establishments offered bicycles and parking spaces to employees close to their place of work (Extended Data Fig. 6, Q24). New and proposed metro rail stations in Chennai, Delhi and Dhaka offer bicycle parking stations. Suburban railway stations in Delhi and Chennai also have paid bicycle parking services near them, enabling last-mile connectivity not just for trains but also for arterial roads. Such stations are actively used by cyclists.

Bicycle repair and parking service providers across the cities mentioned several shared issues, such as taxation, road quality, bicycle designs and increasing economic need to accommodate motorcycle parking and air-filling services. Such service providers, however, appeared to have little political representation as a group or network to lobby for issues concerning the bicycle service economy.

## Discussion

Policy actors in LMICs and globally recognize the importance of increasing bicycling to support a global transition toward sustainable mobility. As we show, in many LMIC cities, supportive infrastructures and home-grown bicycling cultures persist despite increasing automobility and expanding demands to decongest urban roads and build expressways, that is, the portal to foster bicycle cultures has not yet been foreclosed. In contrast to the car-centric built environments in many HICs, LMIC cities navigate a highly evolving vehicleuse culture and infrastructures. The experiences and perspectives of current bicyclists and stakeholders surrounding bicycling cultures offer valuable insights into fostering large increases in bicycling in these environments^23,24,30^, and to decode and interrupt the societal and infrastructural pressures to motorize. Therefore, we used mixed-methods research to understand the current state of and the impediments to bicycling from the perspective of bicycle users and center issues concerning bicycling in four LMIC cities.

Mimi Sheller’s mobility justice framework builds on the idea that all forms of mobilities and their lack are enmeshed in power relations. Sheller uses ‘kinopolitics’ to theorize this dynamic, building on the ‘new mobilities paradigm’^31^, and argues that just mobility transitions require understanding embodied and everyday movement, urban and regional transport systems and global movements (including of knowledge) as interconnected^7^. Our analysis approaches bicycling experiences in Delhi, Chennai, Dhaka and Accra as windows to multiscalar urban kinopolitics—particularly of urban fragmentation—and explores research and policy pathways to mobility justice that the fragments, bicycles, can unveil.

### Bicycling in a splintered city and relational ontologies

Sheller’s kinopolitics manifests in cities through fragmentation: infrastructural developments, such as high-speed road networks, intensify connections to centers of global capital while disconnecting them from local contexts and fracturing urban geographies^32^. Bicyclists in LMICs in our study navigate increasingly fragmented cities, whose geographies are ‘splintered’ along various physical, temporal and institutional axes, including socioeconomic class and migration^7,33^. We find that these bicyclists are predominantly poor, politically disempowered and invisible to most policymakers. In the face of automobile-centric urban growth^34^, bicyclists face systemic marginalization in LMIC cities^35^. Built infrastructural interventions for bicyclists and organized advocacy usually happen in elite parts of the cities aimed at converting car users^36,37^, attending less to the needs of a large fraction of current bicyclists, who tend to be poor, akin to other contexts in Global South^38^, and to the ubiquitous presence of motorized two-wheelers, which are closer to bicycles in cost, form and their use of road space and extant bicycle lanes. Several bicyclists were also domestic migrants to the city and had learnt to bicycle in times or areas with fewer automobiles. The varying bicycle demographics on arterial and residential roads further highlight a gender and age divide. Raised medians, U-turns and multilane arterials physically splinter roads and exacerbate unequal access to bicyclists. Such road features support uninterrupted automobile flow but interrupt and prolong bicycle trips. The few bicycle lanes that remain are, quite literally, in short fragments. The space-time of the city roads was further fragmented through the day and by weather. The fragmentation in and between the roads mirrored institutional splintering. The responsibility for bicycling infrastructure was peppered among different government bodies. Urban development, advocacy and sports affairs agencies that engaged in promoting bicycling amongst youth and automobile users for physical activity benefits and leisure followed choice-based models of physical activity^39^. Foley and colleagues^40^, in the African context, highlight that given the already high levels of walking, policies should focus on context-sensitive improvements to safety and reducing poor walking conditions, rather than simply promoting walking. Following this suggestion, while increasing cycling should remain a goal in LMICs, improving safety for current cyclists, many of whom possess limited financial and political clout, should not be undermined by a focus on converting automobile users. Current, and particularly low-income, bicyclists we met, including the recipients of bicycles distributed by education agencies and philanthropy focused on empowerment through access, faced the consequences of unequal budgetary allocations for roadways, emphasizing unfettered automobility.

While urban automobility splinters cities, studying bicycles as urban fragments, as Colin McFarlane suggests^33^, through the experiences of people who use, repair and retail them, reveals their relations with other elements along urban faultlines. We see that while relationships such as repair services, parking facilities, police assistance and second-hand bicycle access support bicycling, those that deter include dangerous automobile interactions, exertions from physical built environments, safety concerns raised by caregivers and financial barriers to bicycling. Our respondents, even when they individualized the responsibility of risks to the bicyclists, consistently identified traffic safety as a primary concern influencing their choice to bicycle, as has also been reported in HICs^16,41–43^. It is well documented that bicyclists in general face a high risk of injuries in LMICs^44,45^, including owing to caroriented infrastructure that increases exposure for bicyclists^46,47^. However, perceptions and individualized attributions of risk are socially constructed and experienced along the contextually situated axes of power and inequality^48^, here of socioeconomic class. Our findings additionally revealed that relational dynamics in risk perceptions shape the perspectives of adult kin in discouraging children and the elderly from bicycling, extending prior work on age and gender variation in stated preferences against navigating mixed traffic^49^. The broader culture of individualizing such risks has political implications, such that lobbying and public discourse around safer bicycling is largely limited to elite and middle-class-led bicycling organizations. We register a parallel disenfranchisement and lack of organization amongst bicycle repair and parking service providers. However, both elite advocacy for bicycle safety and widespread kin concerns regarding young or elderly bicycling emerged from a framework of care for the potential bicyclists’ health and safety.

Bicyclists’ careful relations and interfaces with other urban elements are important for designing interventions that increase bicycling in ways that resist fragmentation and reconfigure integrated and just mobility systems. Ironically, high-speed automobility creates an environment that is so hazardous for bicycling that the most prominent demand of bicycling advocates and researchers is for bicycle infrastructure that separates them from automobiles. Bicycle lanes and tracks are increasing, albeit slowly, in LMIC cities. However, the utility and value of such physical infrastructure investments will greatly benefit from better geographic understanding of bicyclists’ needs, routes and safety concerns. Bicyclists’ subversions of current road logic particularly offer insights into these, resonating with prior research highlighting the need to audit automobile-enabling road-design features for bicycle friendliness^47,50^.

Beyond hard infrastructure, there is a need to support the ecosystem of material resources, services and stakeholders that enable cycling in LMICs. These are critical relational and collective dimensions of bicycle infrastructures. We observed that existing bicyclists are served by long-standing maintenance infrastructures, including bicycle repair service providers, and resale and reuse cultures thrived across the cities, caring and supporting the longevity of bicycles^51^. By contrast, large bicycling-sharing projects are failing typically because of inadequate maintenance.

**Extended Data Fig. 7:**
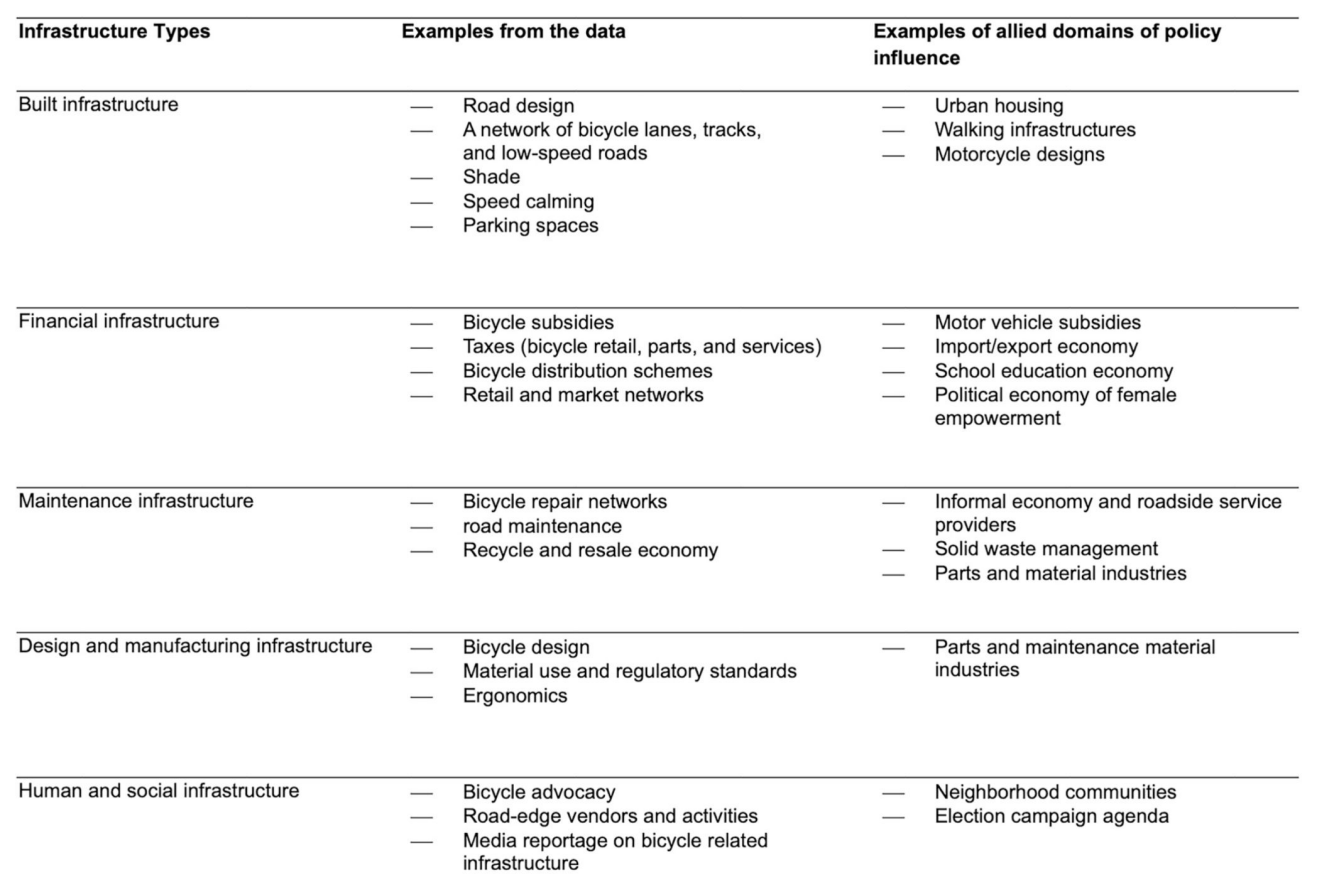
Categorization of Bicycle Infrastructures – Preliminary Themes. Preliminary themes generated from data regarding built, financial, maintenance, design and manufacturing, and human and social infrastructures that support bicycle use and access in the research sites, and their corresponding allied domains of policy influence.

Thus, we argue that bicycling policy must move beyond a fragmented focus on physical infrastructure, especially cycle tracks, and empowerment through bicycle access. An integrated and relational approach, integrating financial, maintenance, planning and human infrastructures (Extended Data Fig. 7), is essential to support bicycling and advance mobility justice in LMIC cities.

### Repairing bicycling infrastructure through an ethics of care

Mobility transitions require recognizing how “differential relations and capabilities for mobility are… built into cultural economies, urban forms, transportation infrastructure, and global mobility regimes, shaping differential access and exposure to harm” (Sheller, p. 436)^31^ and working with an ‘ethics of care’. Policy actors and bicycling stakeholders undertake or partake in caring infrastructures, for instance, through bicycle distribution schemes or letting bicyclists move ahead of vehicular traffic at traffic signals. Even care is far from apolitical. For instance, through an analysis of bicycle lanes in Ecuador, Julie Gamble provides a critical analysis of governmental expressions of care solely through built interventions and crisis infrastructure, which fail to attend to systemic and sociocultural experiences of risk^52^. Alternatively, kin dissuasions of children and the elderly bicycling and bicycle repair work emerge as complex expressions of care in the social and human infrastructures surrounding current and prospective bicyclists.

The limited governmental ‘care’ or scaffolding for bicycling in LMIC cities discussed emerges partly from the perception of vehicles, generally assumed to be automobiles and governed by transportation sectors, as sources of risk that need to be licensed and regulated. As bicycles inherently pose very little risk to their riders compared to motor vehicles, they have largely been ignored from policy perspectives in these cities as personal pursuits. Within the cities’ policy environments, facets of bicycling are governed by education, transportation or sport authorities. However, amid the kinopolitics of automobility, increases in bicycling support public health and sustainable development by reducing greenhouse gas emissions, pollution and traffic injuries, while increasing physical activity. We propose that fostering bicycles for these benefits requires policy advocacy from health and environmental agencies (for example, ministries of health or pollution control agencies), supported by research on the barriers faced by current and prospective bicyclists. While the specific departments implementing or investing in bicycle-related policies may be varied, a coordinating health agency’s care, stewardship and monitoring of enforceable policies can be used to track data-driven implementation to support investments in bicycling.

Learning from bicycle repairers’ care for bicycles and their essential role in supporting bicycle cultures, we adopt a repair-oriented approach to bicycling infrastructures and policy. As Anjaria demonstrates^24^, existing bicycling cultures in these cities challenge perceptions of LMICs as ‘not yet’ having sustainable transportation systems. The cities already engage with bicycling policies and practices, although fragmentarily negotiating heterogeneous and fast-changing transportation environments, as we have demonstrated in this paper. We propose that future policy and research help repair and strengthen existing bicycling infrastructures toward safe, sustainable and just mobility transitions responsive to local needs.

## Methods

We conducted mixed-methods research with interdependent components—ethnographic field observations, intercept surveys and semi-structured interviews^53^. Data collection was led by local teams trained in common methods. The teams maintained ongoing communication to ensure consistency and integration across sites.

**Extended Data Fig. 8:**
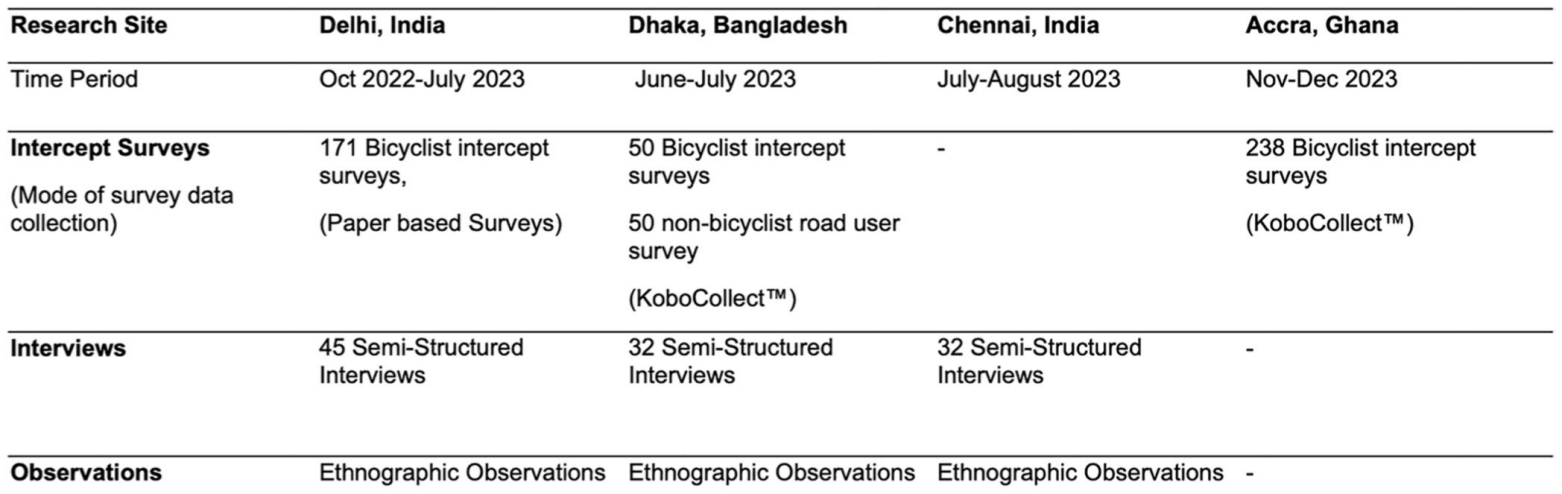
Mixed-Methods Research Activities Conducted. Details and timelines of the mixed-methods research activities conducted in the research sites between October 2022 and December 2023.

Our methodology was geared toward exploring what kinds of data the specific methods and their combinations yield for us. The research activities (Extended Data Fig. 8) were distributed across cities based on the availability of site-specific funding and background data, varying institutional timelines of the research. The research activities were not replicated in all four cities toward a comparative perspective. Instead, we explored what the different methods can offer toward answering the research question and how their insights may be synthesized.

Field observations were conducted to identify research locations (Supplementary Information 1: Ethnographic observation prompt list). Road and market observations undertaken in June and October 2022 in Delhi, Chennai and Dhaka were fluid and grounded in principles of ethnographic research^54^. Observations in June and August 2023 were open-ended, albeit guided by a tentative list of physical infrastructural features, characteristics of cyclist-vehicle interactions, sociodemographic features and any other distinct aspects relevant to bicycling.

Ethnographic research within this project was conducted with cognizance of the multiscalar politics of mobility. The extent of international and intercity engagement in the cities’ fieldwork was limited to the necessities of collaboration and sharing of tools. A mid-project review workshop (internal to the team) and a South Asia-specific stakeholder workshop (public event) were conducted in Delhi. One team member from Chennai participated in the scoping fieldwork in Dhaka. The team members were immersed in local bicycling contexts and general road use in the four cities in various ways throughout the research conception and implementation in 2022–2024. We also paid ongoing attention to emerging bicycling communities, policy changes and urban development processes, including budgetary plans^55–57^, built infrastructures and news stories emerging about cycle infrastructures. Two of the authors commuted to their workplace by bicycle regularly and one participated in city bicycling group events. Many of the authors and research assistants were nonbicyclists themselves but were kin of commuter and leisure bicyclists. Two of the authors bought new bicycles and safety-related accessories during this period, including using a monthly installment financing. In Delhi, Chennai and Dhaka, we made multiple trips to bicycle repair shops to seek services for our bicycles, including air-filling and fixing punctures, and used paid parking services near public transit. We accessed local neighborhood roads, enclaves and arterials, including highways, observing and experiencing bicycling.

Intercept surveys with bicyclists were conducted on arterial roads in Delhi (*N* = 171), Dhaka (*N* = 50) and Accra (*N* = 238), in the local language (Hindi, Bangla, English and Akan). Intercept surveys were conducted where respondents are engaging in the activity being researched^58–60^, allowing focused attention on bicycling sites. Survey sites were chosen to have a high bicyclist presence based on traffic counts in Delhi^61^, field observations, regular transit experiences and information received through interviews in the other cities. Survey respondents (‘respondents’, in this paper) were recruited by researchers on the roadsides of arterial roads. It is important to note that our intercept surveys represent a biased sample of the bicycling population in these cities because of the survey locations (primarily high bicycle volume arterial sites), time of survey (daytime), weather conditions and who agreed to participate. Notably, only some of the bicyclists who rode by the survey site stopped, and this group was probably biased by occupation (and related trip characteristics), gender, age, caste, ethnicity and ability of the bicyclists. Of the very few women that we saw on the arterial roads in Delhi and Dhaka during surveys and field observations, only one (Dhaka) participated in the survey.

Respondents were asked about their most recent trips, income, migration status, household and bicycling skills (Supplementary Information 2: Intercept survey questionnaire). Features of the respondents’ attire and bicycles were noted as a proxy for income, socioeconomic class and employment. Surveys were conducted between October 2022 and November 2023. Additionally, 50 nonbicyclists were surveyed in Dhaka at sites adjacent to the intercept survey sites to explore surveying nonbicyclists who encounter bicycles during road use and to recruit interviewees with varied socioeconomic class, gender and road-use statuses. The focus on bicyclists and stakeholders surrounding them was particular to the 2022–2024 phase of a larger ongoing research project exploring urban bicycling in LMICs, considering funding and project timeline limitations. Our research phase focus on bicyclists, particularly in the intercept surveys, is, by no means, to devalue research with current nonbicyclists in the city regarding bicycling. We see immense potential in such research to explore a range of concerns, including mode shift preferences, safety concerns and travel experiences. The interviews reflect a beginning toward this commitment.

**Extended Data Fig. 9:**
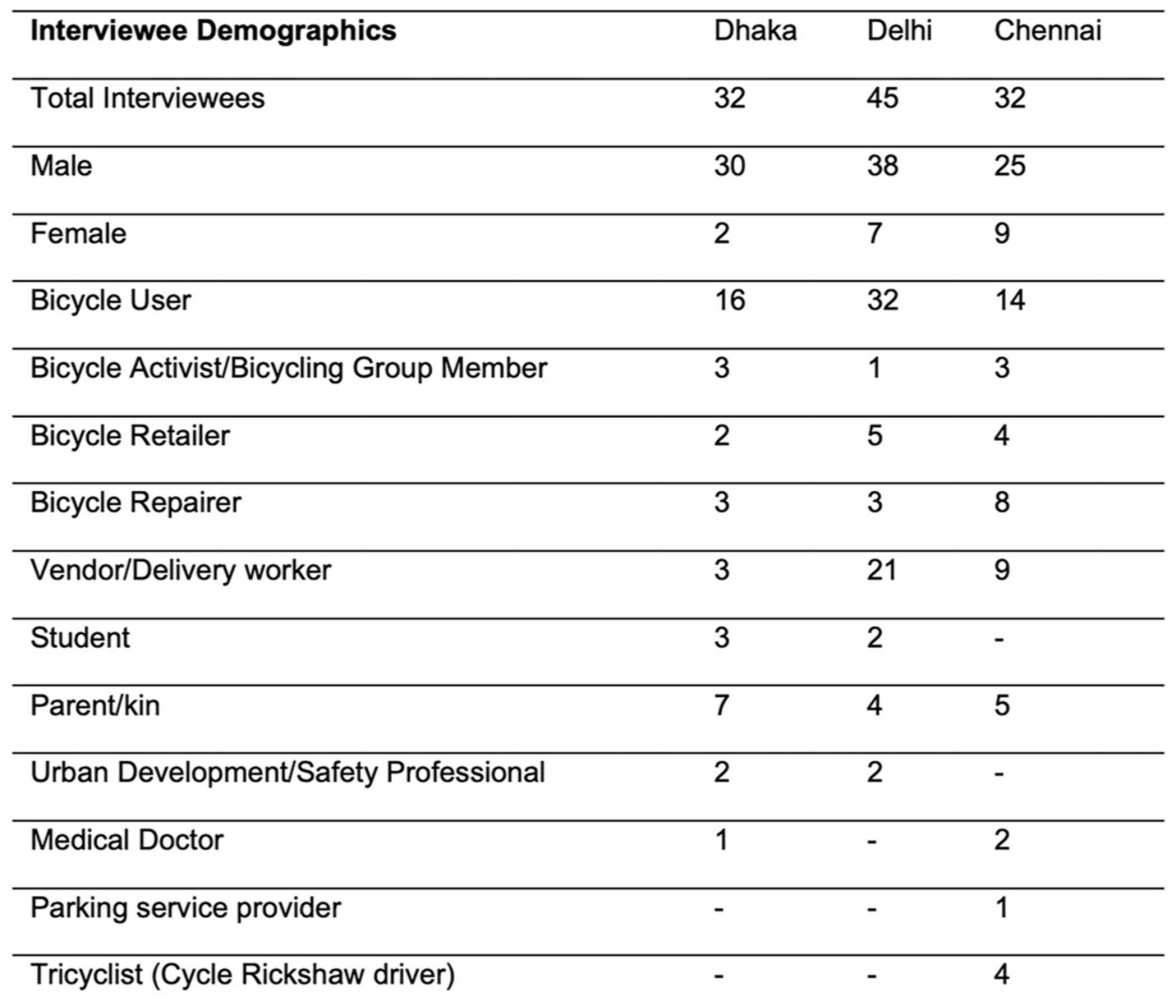
Interviewee Characteristics. The gender, current bicycle use status, and stakeholder role of each interviewee are enumerated and tabulated per city. The numbers listed below only represent audio-recorded semi-structured interviews. Ethnographic interactions and field observations have not been included.

Semi-structured interviews were conducted in Delhi (45), Chennai (32) and Dhaka (32) to explore cyclists’ experiences through in-depth conversation^62^. These were either 8–15-min-long with intercepted cyclists or 20–60-min-long with stakeholders (Supplementary Information 3: Semi-structured interview preliminary questions and probes). Stakeholders (Extended Data Fig. 9) included personnel who either interacted regularly with bicyclists, such as repair service providers and retailers, parking providers or people invested in bicycle infrastructure policy or design, including manufacturers, bicycle activists and officials. Interviews were predominantly conducted in the everyday environments of the users and stakeholders, allowing for conversations regarding their relationship with bicycles, how they use the vehicle and their thoughts on cycling demographics and infrastructure in the cities. Several of the interviewed stakeholders were not currently bicyclists. As indicated in Extended Data Fig. 9, over 50% of the interviewees in Chennai and Dhaka, and 28% in Delhi, were nonbicyclists. In Chennai, interviews were conducted in areas surrounding schools with parents and kin of bicyclists, and two doctors near a school zone. Additionally, we conducted targeted interviews with women bicyclists in Delhi and Chennai because there were very few women who participated in the intercept surveys. We prioritized relational narratives regarding bicycle use and perceptions over personal information, including age, income and other social identifiers.

Interview data and field observation notes were thematically coded using Dedoose in addition to subjective qualitative analysis through discussions with teams across cities. An initial limited list of codes was generated through a literature review. Following open coding of the interview data and ethnographic research, a preliminary list of themes, organized through the lens of infrastructures^25^ (Extended Data Fig. 7), guided data organization. These were further divided into broader themes of transportation equity, sustainability, safety and comfort toward the final synthesis of data. The research employed fluid and responsive constructivist methodologies, policy relevance and field-specific grounded and dialogical thinking among the research team. Policy practicalities and politics of public health-implicated infrastructures are imbricated in the sociopolitical process of producing empirical data and evidence, such as on (1) what counts as bicycle infrastructure and (2) bicycle infrastructure reach, use and limitations. We have attempted both to identify and integrate bicycle infrastructures and allied domains of policy influence through contextual research (Extended Data Fig. 7) and to analyze the findings through the lens of their implementation (Supplementary Information 4: Iterative implementation framework for bicycling in LMIC cities).

Qualitative findings emerged through analytic memo writing and triangulation of survey findings, observations and interview research. We have emphasized analytical depth and relevance of themes over quantitative measures such as frequency of themes or data saturation, in line with established ethnographic research methods^54^. Our decision to do so emerges from the inherently subjective quality of the methods. Further, the time-span of the interview, the interviewer’s understanding of saturation of a theme in a specific interview during the conversation, and across interviews can impact measures such as frequency.

Approaches akin to intersectional analysis were central to our research emphasis on equitable mobility. We studied how gender, class, age and migration intersected in various ways through urban geography in bicyclists’ experiences. One key limitation of our research is that race, ethnicity and caste were not used as frameworks of analysis and require a discussion on the specific geographies of each of the cities, which is beyond the scope of this article.

The study was approved by the Social and Behavioral Sciences Institutional Review Board, The University of Chicago (protocol number IRB22-1171-AM003).

## Extended Data

## Supplementary Material

Supplementary Information

Reporting Summary

## Figures and Tables

**Table 1:** Bicycle use data highlights from intercept surveys

Variable	Mean (range^a^)		
City (no. of respondents)	Delhi (*N*=171)	Dhaka (*N*=50)	Accra (*N*=238)
Age of respondent (years)	39 (19–60)	30 (20–51)	33 (20–64)
Gender (F: M)	(0: 171)	(1: 50)	(0: 238)
Cycling since (age, years)	11.7 (5.6–20)	10 (5.2–19.5)	10.5 (5–20)
Bicycle ownership period (years)	7 (0.6–20)	3.7 (1–16.8)	3.5 (0.25–10)
Cost of bicycle newly	58.4	116.4	77.3
bought (US$)	(10.1–142.2)	(43.7–288.8)	(11.6–190.2)
Cost of second-hand bicycle (US$)	19.6 (7.2–34.5)	39.7 (14.2–83.6)	43.6 (7.6–114.2)
Household income (US$ per month)	216.2	291.8	82.9
	(96.5–663.5)	(91.1–710.8)	(15.3–221.6)^b^
City average household income (US$ per month)	463.4	313.6	399.71
Trip duration (min)	47 (15–90)	30.6 (4–120)	41.1 (5–120)^c^
6-day (weekly) average cycling duration (min)	564	373	493

a (2.5–97.5 percentile); 1 US$ = 82.89 Indian Rupee, 109.73 Bangladeshi Taka and 13.14 Ghanaian Cedis.

b Only informal sector shown (*N* = 153). Of the additional 85 people in the formal sector, 69 earned <US$114 and 16 earned in the range of US$114–228.

c Cycle only or cycle and walk (seven respondents reported mixed travel modes).

**Table 2:** Occupation (percentage) and bicycle use characteristics of intercept survey respondents

Type of work	Dhaka (*N*=37)	Accra (*N*=238)	Delhi (*N*=171)	Bicycle use characteristics
(1) Security guards and peons	0	20	13	Long journeys, fixed schedules, some have odd hours or night shifts
(2) Cleaning staff, domestic helpers, cooks, paid care workers	0	8	5	Multiple stops, fixed day schedules
(3) Factory workers, laborers	5	9	19	Long journey, fixed day schedules, travel together
(4) Delivery workers	16	1	8	Multiple stops, variable day schedules, load carrying
(5) Shop assistants	27	8	3	May do delivery tasks, fixed day schedules, long work hours
(6) Vendors, trade, service providers	41	40	49	Multiple stops, stable bicycles to set up shop, load carrying
(7) Students	0	2	1	Multiple stops to school, college, tuition, travel together
(8) Desk job workers	11	5	2	Health-oriented cycling, fixed day schedules
(9) Unemployed, apprentice, unpaid workers	0	7	1	Multiple stops, care-related travel

Table 2 lists the primary work or occupation reported by the survey respondents. The survey respondents were adult male bicyclists, except for one female bicyclist in Dhaka. The bicycle use characteristics of 2, 4, 6, 7 and 9 include multiple stops and trip chaining, similar to ‘mobilities of care’ and women’s travel described in prior literature on travel and gender^7,16^. Occupations 2, 4–6 and 9 engage in cycling as work^63^. Occupations 1 and 3 were concentrated along the roads connecting low-income housing and institutional areas in the respective cities.

## Data Availability

The tools used in this study are available as supplements to this article. The ethnographic, interview and complete intercept survey data that support the findings of this study cannot be shared owing to confidentiality concerns as they contain potentially identifiable narratives that would compromise participant privacy and the ethical agreements made during the research process. Limited anonymized intercept survey data, with fewer variables, will be shared upon reasonable request to researchers who provide a relevant proposal and agree to data use restrictions designed to protect participant privacy. Requests for access to the limited dataset should be directed to the corresponding author via email. Requesters must submit (1) a brief description of the research purpose, (2) a specification of which variables are required and (3) confirmation of institutional ethics approval or exemption for secondary data analysis. Requests will be reviewed within 4 weeks of receipt, barring exceptional circumstances. Access to controlled data is subject to a data use agreement that prohibits (1) attempts to identify individual participants, (2) linking the dataset with other data sources that could enable re-identification, (3) sharing the data with third parties without prior written approval and (4) use for commercial purposes. Users must agree to report any inadvertent identification of participants and to destroy or return the data upon completion of the approved research project or after 3 years, whichever comes first.

## References

[1] Götschi T, Garrard J, Giles-Corti B (2016). Cycling as a part of daily life: a review of health perspectives. Transp Rev.

[2] Shukla PR, IPCC Climate Change 2022: Mitigation of Climate Change (2022). Contribution of Working Group III to the Sixth Assessment Report of the Intergovernmental Panel on Climate Change.

[3] (2017). Tackling NCDs: Best Buys.

[4] Katzmarzyk PT, Friedenreich C, Shiroma EJ, Lee I-M (2022). Physical inactivity and non-communicable disease burden in low-income, middle-income and high-income countries. Br J Sports Med.

[5] Murray CJL (2020). Global burden of 87 risk factors in 204 countries and territories, 1990–2019: a systematic analysis for the Global Burden of Disease Study 2019. Lancet.

[6] Fuller R (2022). Pollution and health: a progress update. Lancet Planet Health.

[7] Sheller M (2018). Mobility Justice: The Politics of Movement in the Age of Extremes.

[8] Pucher J, Buehler R (2008). Making cycling irresistible: lessons from the Netherlands, Denmark and Germany. Transp Rev.

[9] (2019). Modal Shift in the Boulder Valley: 1990 to 2018.

[10] Thorwaldson L, Thomas F, Carran-Fletcher A (2021). Evaluating the Greenhouse Gas Emission Reduction Benefits from Land Transport Mode Shift Programmes and Projects.

[11] Kärmeniemi M, Lankila T, Ikäheimo T, Koivumaa-Honkanen H, Korpelainen R (2018). The built environment as a determinant of physical activity: a systematic review of longitudinal studies and natural experiments. Ann Behav Med.

[12] Goodman A, Sahlqvist S, Ogilvie D, on behalf of the iConnect Consortium (2014). New walking and cycling routes and increased physical activity: one- and 2-year findings from the UK iConnect Study. Am J Public Health.

[13] Kou Z, Wang X, Chiu SF, Cai H (2020). Quantifying greenhouse gas emissions reduction from bike share systems: a model considering real-world trips and transportation mode choice patterns. Resour Conserv Recycl.

[14] Xu F, Mayuga K (2022). From crisis to opportunity: how the Philippines built 500km of bike lanes in less than a year. World Bank Blogs.

[15] Independent Evaluation Group (2017). Argentina: GEF Sustainable Transport and Air Quality Program.

[16] Ravensbergen L, Buliung R, Laliberté N (2019). Towards feminist geographies of cycling. Geogr Compass.

[17] Lugo AE (2018). Bicycle/Race: Transportation, Culture, & Resistance.

[18] Hoffmann ML (2016). Bike Lanes are White Lanes: Bicycle Advocacy and Urban Planning.

[19] Barajas JM (2021). Biking where Black: connecting transportation planning and infrastructure to disproportionate policing. Transp Res D Transp Environ.

[20] Ravensbergen L (2022). ‘I wouldn’t take the risk of the attention, you know? Just a lone girl biking’: examining the gendered and classed embodied experiences of cycling. Soc Cult Geogr.

[21] Agrawal S, Seth A, Goel R (2024). A silent revolution: rapid rise of cycling to school in rural India. J Transp Geogr.

[22] Muralidharan K, Prakash N (2017). Cycling to school: increasing secondary school enrollment for girls in India. Am Econ J Appl Econ.

[23] Goel R (2023). Gender gap in mobility outside home in urban India. Travel Behav Soc.

[24] Anjaria JS (2024). Mumbai on Two Wheels: Cycling, Urban Space and Sustainable Mobility.

[25] Larkin B (2013). The politics and poetics of infrastructure. Annu Rev Anthropol.

[26] Yu W (2024). Estimates of global mortality burden associated with short-term exposure to fine particulate matter (PM2.5). Lancet Planet Health.

[27] Goel R (2022). Cycling behaviour in 17 countries across 6 continents: levels of cycling, who cycles, for what purpose, and how far?. Transp Rev.

[28] Dhar S, Shukla PR, Pathak M (2017). India’s INDC for transport and 2°C stabilization target. Chem Eng Trans.

[29] Bhan G (2013). Planned illegalities: housing and the ‘failure’ of planning in Delhi: 1947–2010. Econ Polit Wkly.

[30] Silonsaari JE (2024). Prefigurative politics in action research for just cycling futures. Urban Plan Transp Res.

[31] Sheller M (2023). Mobility justice after climate coloniality: mobile commoning as a relational ethics of care. Aust Geogr.

[32] Graham S, Marvin S (2009). Splintering Urbanism: Networked Infrastructures, Technological Mobilities and the Urban Condition.

[33] McFarlane C (2018). Fragment urbanism: politics at the margins of the city. Environ Plan Soc Space.

[34] Gopakumar G (2020). Installing Automobility: Emerging Politics of Mobility and Streets in Indian Cities.

[35] Joshi R, Joseph Y (2015). Invisible cyclists and disappearing cycles. Transfers.

[36] Joshi R, Baby J, Norcliffe G (2022). Routledge Companion to Cycling.

[37] Anantharaman M (2017). Elite and ethical: the defensive distinctions of middle-class bicycling in Bangalore, India. J Consum Cult.

[38] Power D, Glaser M, Gemerts R, Lartey D, Soares BO (2025). How to ‘de-Dutch’ the bicycle: development of active mobility policy on the Caribbean island of Bonaire through real-world mixed methods research. Transp Res Interdiscip Perspect.

[39] Salvo D, Jáuregui A, Adlakha D, Sarmiento OL, Reis RS (2023). When moving is the only option: the role of necessity versus choice for understanding and promoting physical activity in low- and middle-income countries. Annu Rev Public Health.

[40] Foley L (2022). Socioeconomic and gendered inequities in travel behaviour in Africa: mixed-method systematic review and meta-ethnography. Soc Sci Med.

[41] Huber S, Lindemann P, Schröter B (2023). Understanding the influence of accident risk and perceived safety on bicycle route choice. Transp Res Procedia.

[42] Rivera Olsson S, Elldér E (2023). Are bicycle streets cyclist-friendly? Micro-environmental factors for improving perceived safety when cycling in mixed traffic. Accid Anal Prev.

[43] Aldred R, Woodcock J, Goodman A (2016). Does more cycling mean more diversity in cycling?. Transp Rev.

[44] Scarano A, Aria M, Mauriello F, Riccardi MR, Montella A (2023). Systematic literature review of 10 years of cyclist safety research. Accid Anal Prev.

[45] Olivier J, Creighton P (2016). Bicycle injuries and helmet use: a systematic review and meta-analysis. Int J Epidemiol.

[46] Goel R (2023). Population-level estimate of bicycle use and fatality risk in a data-poor setting. Int J Inj Contr Saf Promot.

[47] Paturi R, Agrawal S, Bilam S, Bhalla K, Goel R (2024). Case–control study of fatal bicycle crashes in peri-urban areas of Delhi. Inj Prev.

[48] Lupton D (2023). Risk.

[49] Aldred R, Elliott B, Woodcock J, Goodman A (2017). Cycling provision separated from motor traffic: a systematic review exploring whether stated preferences vary by gender and age. Transp Rev.

[50] Xiao CS (2023). Design effects of cycle infrastructure changes: an exploratory analysis of cycle levels. Transp Res Interdiscip Perspect.

[51] Anwar NH, Sur M (2020). Keeping cities in motion: an introduction to the labours of repair and maintenance in South Asia. Econ Polit Wkly.

[52] Gamble J (2024). Rearranging care while cycling under the COVID-19 pandemic in Quito, Ecuador. Gend Place Cult.

[53] Schoonenboom J, Johnson RB (2017). How to construct a mixed methods research design. Kölner Z Soz Sozialpsychol.

[54] Ladner S (2014). Practical Ethnography: A Guide to Doing Ethnography in the Private Sector.

[55] Finance Department, Government of National Capital Territory of Delhi (2025). Budget at a Glance 2025–2026.

[56] (2023). Grant No.34. 137—Local Government Division.

[57] (2025). Composite Budget for 2025–2028 Programme Based Budget Estimates for 2025.

[58] Transportation Research Board, National Academies of Sciences, Engineering, and Medicine & Schaller, B (2005). On-Board and Intercept Transit Survey Techniques.

[59] Ojo TK (2022). An intercept survey of the use and non-use of footbridges in Ghana. Case Stud Transp Policy.

[60] Miller KW, Wilder LB, Stillman FA, Becker DM (1997). The feasibility of a street-intercept survey method in an African– American community. Am J Public Health.

[61] Malik L, Tiwari G, Khanuja RK Suresh (2020). Classified Traffic Volume and Speed Study Delhi (2018) TRIPP-PR-20-01.

[62] Wengraf T (2001). Qualitative Research Interviewing: Biographical Narrative and Semi-Structured Methods.

[63] Murthy M, Sur M (2023). Cycling as work: mobility and informality in Indian cities. Mobilities.

[64] Largest Cities by Population 2025.

[65] United Nations Habitat Urban Data (2025). Open Spaces and Green Areas.

[66] (2025). 2024 World Air Quality Report Region and City PM2.5 Ranking.

[67] (2022). Road Accidents in India 2022.

[68] (2022). Accra Road Safety Report 2022.

[69] Johns Hopkins International Injury Research Unit (2023). Round 1–3 Project Report on Road Safety Risk Factors in Dhaka North.

[70] (2020). Baseline Report: Enabling Strategic Plan: Master Plan for Delhi 2041.

[71] CMDA, CMRL & UMTC (2019). Comprehensive Mobility Plan for Chennai Metropolitan Area: Final Report—May 2019.

[72] Neta M, Martin P, Hoyez M, Grant Monney M, Teyvi A (2023). Ghana Mobility Study–SPTA: Final Report: Market Potential, Clients and User Groups for New Mobility Services in Ghana.

[73] Noor-E-Alam (2018). Final Report on Sustainable Urban Transport Index (SUTI) for Dhaka, Bangladesh.

[74] United Nations Habitat Urban Data (2025). Urban Transport.

[75] Ramachandra TV, Aithal BH, Sreejith K (2015). GHG footprint of major cities in India. Renew Sustain Energy Rev.

[76] Mishra S (2022). Whose lane is it anyway? Why cyclists in Delhi find selves in no man’s land. Times of India.

[77] Hossain S (2023). Paved for pedals, occupied by vehicles, vendors. The Daily Star.

