# Supplementary material for: The status and politics of bicycling in the cities of low- and middle-income countries
Source: Nat Cities. Author manuscript; Available in PMC 2026 Feb 21. (PMC7618767; doi:10.1038/s44284-025-00367-y)
Supplement: Supplementary Information [file EMS212293-supplement-Supplementary_Information.pdf]

Supplementary information The online version contains supplementary material available at https://doi.org/10.1038/s44284-025-00367-y.
