# Supplementary material for: The status and politics of bicycling in the cities of low- and middle-income countries
Source: Nat Cities. Author manuscript; Available in PMC 2026 Feb 21. (PMC7618767; doi:10.1038/s44284-025-00367-y)
Supplement: Reporting Summary [file EMS212293-supplement-Reporting_Summary.pdf]

nature portfolio

Corresponding author(s): Smruthi Bala Kannan

Last updated by author(s): Oct 11, 2025

Reporting Summary

Nature Portfolio wishes to improve the reproducibility of the work that we publish. This form provides structure for consistency and transparency in reporting. For further information on Nature Portfolio policies, see our Editorial Policies and the Editorial Policy Checklist.

Statistics

For all statistical analyses, confirm that the following items are present in the figure legend, table legend, main text, or Methods section.

n/a	Confirmed	
☐	☒	The exact sample size (*n*) for each experimental group/condition, given as a discrete number and unit of measurement
☐	☒	A statement on whether measurements were taken from distinct samples or whether the same sample was measured repeatedly
☒	☐	The statistical test(s) used AND whether they are one- or two-sided<br>*Only common tests should be described solely by name; describe more complex techniques in the Methods section.*
☒	☐	A description of all covariates tested
☒	☐	A description of any assumptions or corrections, such as tests of normality and adjustment for multiple comparisons
☐	☒	A full description of the statistical parameters including central tendency (e.g. means) or other basic estimates (e.g. regression coefficient) AND variation (e.g. standard deviation) or associated estimates of uncertainty (e.g. confidence intervals)
☒	☐	For null hypothesis testing, the test statistic (e.g. *F*, *t*, *r*) with confidence intervals, effect sizes, degrees of freedom and *P* value noted<br>*Give P values as exact values whenever suitable.*
☒	☐	For Bayesian analysis, information on the choice of priors and Markov chain Monte Carlo settings
☒	☐	For hierarchical and complex designs, identification of the appropriate level for tests and full reporting of outcomes
☒	☐	Estimates of effect sizes (e.g. Cohen's *d*, Pearson's *r*), indicating how they were calculated

Our web collection on statistics for biologists contains articles on many of the points above.

Software and code

Policy information about availability of computer code

Data collection	KoboToolBox was used for survey data collection (kobotoolbox.org) in Dhaka (Bangladesh) and Accra (Ghana)
Data analysis	Dedoose was used for qualitative interview data analysis (dedoose.com)

For manuscripts utilizing custom algorithms or software that are central to the research but not yet described in published literature, software must be made available to editors and reviewers. We strongly encourage code deposition in a community repository (e.g. GitHub). See the Nature Portfolio guidelines for submitting code & software for further information.

Data

Policy information about availability of data

All manuscripts must include a data availability statement. This statement should provide the following information, where applicable:

- Accession codes, unique identifiers, or web links for publicly available datasets
- A description of any restrictions on data availability
- For clinical datasets or third party data, please ensure that the statement adheres to our policy

The tools used in this study are available as supplements to this paper. The ethnographic, interview, and complete intercept survey data that support the findings of this study cannot be shared due to confidentiality concerns, as they contain potentially identifiable narratives that would compromise participant privacy and the ethical agreements made during the research process. Limited anonymized intercept survey data, with fewer variables, will be shared upon reasonable request to researchers who provide a relevant proposal and agree to data use restrictions designed to protect participant privacy.

Requests for access to the limited dataset should be directed to the corresponding author via email. Requesters must submit: (1) a brief description of the research purpose, (2) a specification of which variables are required, and (3) confirmation of institutional ethics approval or exemption for secondary data analysis. Requests will be reviewed within four weeks of receipt, barring exceptional circumstances.

Access to controlled data is subject to a data use agreement that prohibits: (1) attempts to identify individual participants, (2) linking the dataset with other data sources that could enable re-identification, (3) sharing the data with third parties without prior written approval, and (4) use for commercial purposes. Users must agree to report any inadvertent identification of participants and to destroy or return the data upon completion of the approved research project or after 3 years, whichever comes first.

Research involving human participants, their data, or biological material

Policy information about studies with human participants or human data. See also policy information about sex, gender (identity/presentation), and sexual orientation and race, ethnicity and racism.

Reporting on sex and gender	Gender was considered in the study design. Participant gender was determined based on self-reporting and culturally relevant presentation. Consent was obtained to report anonymized individual level data. Gender distribution of the intercept survey samples have been presented in the paper.
Reporting on race, ethnicity, or other socially relevant groupings	Socio-economic status and age have been used. Socio-economic status was determined based on self-reported household income and vehicle ownership collected in the intercept survey, and income, employment, and vehicle ownership discussed in semi-structured interviews. Social perceptions of class, age, and geography were discussed.
Population characteristics	See above
Recruitment	The participants were recruited through interactions in public spaces. The recruiting teams were mixed gender to ensure that both men and women were comfortable with responding to the research interaction.
Ethics oversight	Social and Behavioral Sciences Institutional Review Board, The University of Chicago (Protocol number: IRB22-1171-AM003)

Note that full information on the approval of the study protocol must also be provided in the manuscript.

Field-specific reporting

Please select the one below that is the best fit for your research. If you are not sure, read the appropriate sections before making your selection.

☐ Life sciences ☒ Behavioural & social sciences ☐ Ecological, evolutionary & environmental sciences

For a reference copy of the document with all sections, see nature.com/documents/nr-reporting-summary-flat.pdf

Behavioural & social sciences study design

All studies must disclose on these points even when the disclosure is negative.

Study description	Mixed methods study: Intercept survey (quantitative) and interview and ethnographic studies (qualitative)
Research sample	Bicyclists and stakeholders in bicycling in India, Ghana, and Bangladesh. Adults (Over the age 18). The rationale for the choice of research sample is its direct connection to the research question.
Sampling strategy	This was an exploratory research where sample size was not predetermined. Final sample sizes of the intercept surveys and interview are stated in the main article. Our sample is not representative and no sample size calculation was performed. We did purposive sampling of the sites with high bicyclist presence where we approached bicyclists based on the research team's prior research of the city's demographic spread and traffic patterns (Traffic counts in Delhi, field observations, regular transit experiences, and information received through interviews in the other cities) . Thematic saturation of data was considered aligned with qualitative data analysis as described in the article.
Data collection	All the data collection was conducted in public spaces such as roadsides, parks, and storefronts. Data collection was led by local teams trained in common methods. The teams maintained ongoing communication to ensure consistency and integration across sites. Intercept Surveys were recorded pen on paper in Delhi (India), and using software KoboCollect (KoboToolBox) in Dhaka (Bangladesh) and Accra (Ghana). Interviews were audio recorded or notes taken pen on paper. The field observations of the ethnographic study were collected through researcher notes and some photos and videos of public sites. The participants were informed of the study purpose and focus.<br><br>This exploratory mixed methods research involves researchers' full awareness and understanding of the research questions and attention to context. The researchers explained the research intent and questions to the participants during informed consent. Since the research was conducted in public spaces, people apart from the researcher and the participant were present at varying distances from the research interactions. Appropriate terms of anonymity or conversation were situationally negotiated with context awareness and sensitivity.
Timing	The research was conducted between October 2022 and November 2023.

Data exclusions	No data was excluded from analysis.
Non-participation	The recruitment for the human subject interactions — intercept survey and semi-structured interviews — were conducted on roadsides and public sites. Only bicyclists and road-users interested in participation stopped to respond to the intercept survey. This group was likely biased by factors such as occupation (and related trip characteristics), gender, age, caste, ethnicity, and ability. The number of people surveyed was also limited by the research duration and the time of research during the day. Of the very few women that we saw on the arterial roads in Delhi and Dhaka during surveys and field observations, only one (Dhaka) participated in the survey.
Randomization	Bicyclists who used arterial and non-arterial roads during different times of the day were approached. We did not conduct surveys at night for researcher safety. We varied the sites of research within each city to interact with men and women, with varying socio-economic statuses.

Reporting for specific materials, systems and methods

We require information from authors about some types of materials, experimental systems and methods used in many studies. Here, indicate whether each material, system or method listed is relevant to your study. If you are not sure if a list item applies to your research, read the appropriate section before selecting a response.

Materials & experimental systems

n/a	Involved in the study
☒	☐ Antibodies
☒	☐ Eukaryotic cell lines
☒	☐ Palaeontology and archaeology
☒	☐ Animals and other organisms
☒	☐ Clinical data
☒	☐ Dual use research of concern
☒	☐ Plants

Methods

n/a	Involved in the study
☒	☐ ChIP-seq
☒	☐ Flow cytometry
☒	☐ MRI-based neuroimaging

Plants

Seed stocks	Not Applicable
Novel plant genotypes	Not Applicable
Authentication	Not Applicable
